# Association between Body Mass Index and Left Ventricular Ejection Fraction in Obese Patients with Atrial Fibrillation Referred for Catheter Ablation

**DOI:** 10.3389/fnut.2025.1712935

**Published:** 2026-01-12

**Authors:** Rong Wan, Yuhao Su, Ying Huang

**Affiliations:** 1 Jiangxi Key Laboratory of Molecular Medicine, Nanchang University, Nanchang, Jiangxi, China; 2 Cardiovascular Department, The Second Affiliated Hospital of Nanchang University, Nanchang, Jiangxi, China; 3 Rehabilitation Department, The Second Affiliated Hospital, Jiangxi Medical College, Nanchang University, Nanchang, Jiangxi, China

**Keywords:** atrial fibrillation, ejection fraction, heart failure, linear regression, obesity

## Abstract

**Background:**

Atrial fibrillation (AF) is one of the most common arrhythmias associated with obesity and metabolic disorders. However, little is known about the association between body mass index (BMI) and left atrial size and left ventricular ejection fraction (LVEF) in AF patients referred for catheter ablation.

**Methods:**

We retrospectively obtained a dataset (*N* = 170) from the Dryad database, in which the association between alcohol consumption, cardiac biomarkers, left atrial size, and re-ablation in patients with AF referred for catheter ablation was analyzed as part of the SMURF (Symptom burden, Metabolic profile, Ultrasound findings, Rhythm, neurohormonal activation, haemodynamics and health-related quality of life in patients with atrial Fibrillation) study. Multivariable linear regression models were used to investigate the association between baseline BMI and left atrial size and LVEF in these patients.

**Results:**

Correlation analysis displayed that BMI was negatively associated with LVEF, but it had no correlation with left atrial volume (LAV)/body surface area (BSA). When evaluating BMI as an independent variable, our linear regression analysis for adjusted coefficient of LVEF [correlation coefficient = −0.46, 95% CI: (−0.78, −0.15), *p* = 0.0049] was very significant after age, gender, smoking, alcohol consumption and estimated glomerular filtration rate (eGFR) were controlled for. However, the correlation coefficients for LAVmax/BSA and LAVmin/BSA were −0.01 and 0.13, respectively, and were not statistically significant after adjusting for the same covariates.

**Conclusion:**

We observed that BMI had a significantly negative correlation with LVEF but not with LAVmax/BSA or LAVmin/BSA in the obese patients with AF referred for catheter ablation. This suggests that obesity may be associated with an increased risk of impaired cardiac contractile function in these patients.

## Introduction

Obesity has been confirmed to be significantly associated with various cardiovascular and metabolic diseases, including hypertension, diabetes, coronary heart disease, heart failure, stroke, and dyslipidemia (1–4). An “obesity paradox” has also been observed in most cardiovascular diseases (CVDs), whereby individuals who are overweight or mildly obese tend to have a better prognosis than those who are of normal weight or underweight (1, 2). Correspondingly, this contradiction has also been observed in the relationship between obesity and atrial fibrillation (AF), a common disease in older adults with serious complications, including cardiogenic stroke, heart failure, and even myocardial infarction (MI) (1, 2). Several previous studies, for instance, have reported a strong association between high adiposity, as measured by body mass index (BMI), and the risk of persistent and post-ablation AF (3, 4). Site-specific fat, including epicardial and visceral adiposity, has also shown a consistent association with a higher risk of AF progression and recurrence. In contrast to these previous findings, some clinical studies have suggested that overweight or obesity is negatively associated with all-cause mortality during long-term follow-up (5–7). Pandey et al. reported that AF patients with class I obesity had a 35% lower risk of all-cause mortality compared to those with normal BMI in a cohort study of patients with prevalent AF from the Outcomes Registry for Better Informed Treatment of Atrial Fibrillation (ORBIT-AF) registry (5). Previous secondary analyses from the Atrial Fibrillation Follow-Up Investigation of Rhythm Management (AFFIRM) study (6) and the Apixaban for Reduction in Stroke and Other Thromboembolic Events in Atrial Fibrillation (ARISTOTLE) study (7) have also confirmed similar results. Recently, a large meta-analysis including eight such cohort studies further confirmed the presence of an “obesity paradox,” showing that patients with AF and higher body weight had more favorable prognoses (8). The specific physiological or pathological mechanisms underlying the contradiction are not fully understood. However, it can be clearly stated that the enlargement and functional decline of the heart caused by AF are important pathological factors leading to adverse cardiovascular events (3–8). Nonetheless, the potential associations between obesity and left ventricular ejection fraction (LVEF), as well as atrial size, in AF patients remain unclear.

In this study, we obtained raw data from the Dryad database, in which the association between alcohol consumption, cardiac biomarkers, left atrial size, and re-ablation in AF patients referred for catheter ablation was analyzed as part of the SMURF (Symptom burden, Metabolic profile, Ultrasound findings, Rhythm, neurohormonal activation, haemodynamics and health-related quality of life in patients with atrial Fibrillation) study. Our objective was to further evaluate the associations between BMI and left atrial size and LVEF in AF patients referred for catheter ablation (*N* = 170).

## Materials and Methods

### Study population

Our data were collected from the SMURF study, of which a partial subset (*N* = 192) is stored in the Dryad database^1^ (9). The protocol of the SMURF study has been published previously (10). In compliance with the Declaration of Helsinki, the study (Dnr 2011/40-31, 2012/226-32) was approved by the Regional Ethical Review Board in Linkoping, Sweden, and all included participants provided written informed consent (11). In summary, the study was conducted in patients referred for radiofrequency ablation due to AF at the University Hospital in Linkoping, Sweden, between January 2012 and April 2014. Each enrolled patient was invited to complete a comprehensive baseline evaluation, including demographic characteristics, lifestyle factors, medical history, physical examination, and electrocardiogram (ECG). Transthoracic echocardiography (TTE) was performed the day before radiofrequency ablation, after completion of the baseline questionnaires. Blood samples were also collected from the femoral vein of each patient for biomarker analysis before the ablation. All patients underwent catheterization according to clinical routine on the day of the ablation procedure. The ablation procedure has been described previously (9, 10). The inclusion criteria were as follows: (1) age over 18 years with persistent or paroxysmal AF, (2) patients who underwent radiofrequency ablation for the first time, and (3) patients who could complete the study questionnaires. The exclusion criteria were as follows: (1) Patients scheduled for heart surgery or with a history of heart surgery, (2) patients who had previously undergone catheter or surgical AF ablation, (3) patients with an LVEF less than 35%, and (4) patients who had experienced acute coronary syndrome within the past 3 months. BMI was calculated as weight (kg)/square of height (m^2^). After excluding 22 patients with missing data from the Dryad database, a total of 170 patients were included in our analysis.

### Echocardiography

All patients underwent TTE using the GE Vivid 7 or GE Vivid E9 system (GE Healthcare, Horten, Norway) before the ablation procedure. The Simpson’s biplane method was used to calculate LVEF. Left atrial volume (LAV) was calculated using the biplane area-length method (12). The left atrial volume (LAV) index was calculated as left atrial volume (LAV)/body surface area (BSA).

### Definition of covariates

Chronic kidney disease (CKD) was defined as an estimated glomerular filtration rate (eGFR) of less than 60 mL/min/1.73 m^2^ (13). Previous smokers were defined as individuals who had smoked at any point in the past. Sex was categorized as female and male. Alcohol consumption was defined as self-reported intake (units/week). Medical history, including hypertension, diabetes mellitus, stable angina, previous MI, heart failure, previous stroke or transient ischemic attack (TIA), peripheral arterial disease (PAD), and CKD, was recorded as “yes” or “no” for each condition. The CHA2DS2VASc was defined as the total score of congestive heart failure, hypertension, age ≥75, diabetes, stroke/TIA, vascular disease, age 65–74 and sex category.

### Laboratory indicators

Blood concentrations of atrial natriuretic peptide (ANP), B-type natriuretic peptide (BNP), cholesterol, triglycerides (TG), low-density lipoprotein (LDL), and high-density lipoprotein (HDL) were measured as described previously (13). Aspartate aminotransferase (AST) and alanine aminotransferase (ALT) were determined according to the biochemical examination of clinical routine. Other laboratory indicators were also described in detail previously (9, 10, 13).

### Statistical analysis

All statistical analyses were performed using Empower version 4.1, and a *p*-value of ≤0.05 was considered statistically significant. Clinical characteristics were presented as percentages for categorical variables and as mean ± standard deviations (SD) for continuous variables. We classified all participants into two subgroups according to the presence of heart failure (heart failure and non-heart failure groups) and then used a linear regression model to investigate the associations between BMI value and LVEF and LAV/BSA among these AF patients. The correlation coefficient and 95% confidence interval (CI) were calculated separately for the two subgroups. We included age, sex, smoking, alcohol consumption, and eGFR as potential confounders. Model 1, assessing the associations between BMI and LVEF and LAV/BSA, was adjusted for age and sex. Model 2 was adjusted for age, sex, smoking, alcohol consumption, and eGFR.

To further solve the possibility of confounding effect for the observed associations, sensitivity analysis was used to further exclude ANP and BNP for the independent association. ANP and BNP are important biomarkers of cardiac dysfunction which are confounding variables affecting the results. Therefore, we further excluded the influence of the two covariates on the independent association through our sensitivity analysis. Finally, we conducted a separate analysis to calculate correlation coefficients and 95% CIs for the associations between baseline BMI and LVEF and LAV in the AF patients, stratified by medical history, including hypertension and diabetes mellitus.

## Results

### Clinical characteristics of the AF patients

**Figure 1:**
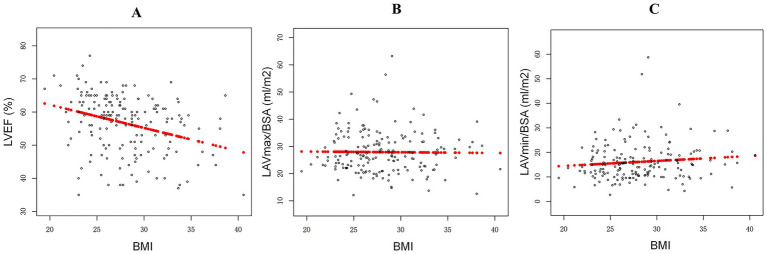
Associations between body mass index (BMI) and left ventricular ejection fraction (LVEF) **(A)** and left atrial volume/body surface area (LAV/BSA) **(B,C)** in patients with atrial fibrillation.

Among the 170 participants, the mean age was 60.31 ± 10.28 years, and 49 (28.82%) individuals were female, as shown in Table 1. The LVEF, LAVmax/BSA, and LAVmin/BSA in these AF patients were 56.59 ± 8.85, 27.91 ± 7.78 mL/m^2^, and 16.10 ± 7.74 mL/m^2^, respectively. The mean BMI of the participants was 28.01 ± 4.02 kg/m^2^. The prevalence of medical history conditions was as follows: hypertension, 72 (42.4%); diabetes mellitus, 13 (7.6%); stable angina, 1 (0.6%); previous myocardial infarction (MI), 9 (5.3%); heart failure, 17 (10.0%); previous stroke or TIA, 15 (8.8%); and PAD, 1 (0.6%). As shown in Figures 1A–C, our univariate correlation analysis showed that higher BMI was significantly associated with reduced LVEF (*p* < 0.05), but it was not correlated with LAVmax/BSA or LAVmin/BSA (all *p* > 0.05).

**Table 1:** Admission characteristics in patients with AF (*N* = 170).

Variables	Total patients (*N* = 170)
Age (years)	60.31 ± 10.28
Gender (female), *n* (%)	49 (28.82%)
Body mass index (kg/m^2^)	28.01 ± 4.02
Alcohol consumption (units/week)	5.83 ± 6.60
Previous smokers, *n* (%)	84 (49.41%)
LVEF (%)	56.59 ± 8.85
LAVmax/BSA (mL/m^2^)	27.91 ± 7.78
LAVmin/BSA (mL/m^2^)	16.10 ± 7.74
Systolic blood pressure (mmHg)	145.78 ± 20.36
Diastolic blood pressure (mmHg)	89.86 ± 11.28
CHA2DS2VASc	
0	48 (28.24%)
1	36 (21.18%)
2	39 (22.94%)
3	27 (15.88%)
4	17 (10.00%)
5	2 (1.18%)
6	1 (0.59%)
Medical history	
Hypertension, *n* (%)	72 (42.35%)
Stable angina, *n* (%)	1 (0.59%)
Previous MI, *n* (%)	9 (5.29%)
Diabetes mellitus, *n* (%)	13 (7.65%)
PAD, *n* (%)	1 (0.59%)
Heart failure, *n* (%)	17 (10.00%)
Previous stroke or TIA, *n* (%)	15 (8.82%)
Laboratory measurements	
CKD (eGFR < 60 mL/min/1.73 m^2^)	33 (19.41%)
ALT (μkat/L)	0.50 ± 0.23
AST (μkat/L)	0.47 ± 0.14
LDL (mmol/L)	3.22 ± 1.02
HDL (mmol/L)	1.23 ± 0.35
TG (mmol/L)	1.24 ± 0.61
ANP (pg/mL)	151.99 ± 81.61
BNP (pg/mL)	403.23 ± 572.12

AF, atrial fibrillation; LVEF, left ventricular ejection fraction; MI, myocardial infarction; PAD, peripheral arterial disease; TIA, transient ischemic attack; eGFR, estimated glomerular filtration rate; ALT, alanine aminotransferase; AST, aspartate aminotransferase; LDL, low density lipoprotein; HDL, high density lipoprotein; TG, triglyceride; ANP, atrial natriuretic peptide; BNP, brain natriuretic peptide; CHA2DS2VASc, congestive heart failure, hypertension, age ≥ 75, diabetes, stroke/TIA, vascular disease, age 65–74, sex category; LAV/BSA, left atrial volume/body surface area.

### The associations between BMI and LVEF and LAV in the AF patients

When evaluating BMI as an independent variable and LVEF and LAV as dependent variables, the multivariable-adjusted correlation coefficient for LVEF was −0.46 [95% CI: (−0.78, −0.15), *p* = 0.0049; Model 2] and was statistically significant after adjusting for age, sex, smoking, alcohol consumption, and eGFR (as shown in Table 2). However, the correlation coefficients for LAVmax/BSA and LAVmin/BSA in Model 2 were −0.01 and 0.13, respectively, and were not statistically significant (all *p* > 0.05) after adjusting for these covariates. Furthermore, we observed a significant difference related to heart failure: the significant association between BMI and LVEF was present in the patients without heart failure (correlation coefficient = −0.57; 95% CI: −0.92, −0.22, *p* = 0.0016) but not in those with heart failure (correlation coefficient = 0.21; 95% CI: −0.64, 1.06, *p* = 0.6402). Regarding the association between BMI and LAV, no significant relationship was observed in either patients with or without heart failure.

**Table 2:** The linear regression model for the association between BMI value on admission and LVEF and LAV/BSA in AF patients.

Variables	Model 1		Model 2	
	Correlation coefficient (95% CI)	*p*-value	Correlation coefficient (95% CI)	*p*-value
LVEF				
Total patients (*N* = 170)	−0.51 (−0.81, −0.22)	0.0009	−0.46 (−0.78, −0.15)	0.0049
Patients with heart failure (*N* = 17)	−0.03 (−0.82, 0.76)	0.9399	0.21 (−0.64, 1.06)	0.6402
Patients without heart failure (*N* = 153)	−0.60 (−0.92, −0.28)	0.0004	−0.57 (−0.92, −0.22)	0.0016
LAVmax BSA				
Total patients (*N* = 170)	−0.04 (−0.34, 0.25)	0.7703	−0.01 (−0.32, 0.31)	0.9595
Patients with heart failure (*N* = 17)	−0.69 (−1.94, 0.56)	0.2974	−0.14 (−1.18, 0.91)	0.8047
Patients without heart failure (*N* = 153)	−0.00 (−0.30, 0.30)	0.9915	0.01 (−0.31, 0.33)	0.9470
LAVmin BSA				
Total patients (*N* = 170)	0.13 (−0.15, 0.42)	0.3614	0.13 (−0.17, 0.44)	0.3920
Patients with heart failure (*N* = 17)	−0.38 (−1.76, 1.00)	0.6011	0.03 (−1.25, 1.31)	0.9668
Patients without heart failure (*N* = 153)	0.16 (−0.12, 0.44)	0.2681	0.14 (−0.16, 0.45)	0.3572

Model 1, adjusted for age and gender.

Model 2, adjusted for age, gender, smoking, alcohol consumption and eGFR.

BMI, body mass index; AF, atrial fibrillation; LVEF, left ventricular ejection fraction; LAV/BSA, left atrial volume/body surface area; eGFR, estimated glomerular filtration rate.

Moreover, sensitivity analyses were conducted to address the potential confounding effects on the independent association (Table 3). We observed little change in the relationship between BMI and LVEF after including covariates such as ANP and BNP in the multivariable-adjusted models.

**Table 3:** Sensitivity analysis of linear regression model.

Variables	Model 1		Model 2	
	Correlation coefficient (95% CI)	*p*-value	Correlation coefficient (95% CI)	*p*-value
LVEF				
Total patients (*N* = 170)	−0.48 (−0.75, −0.20)	0.0007	−0.43 (−0.73, −0.14)	0.0045
Patients with heart failure (*N* = 17)	−0.04 (−0.90, 0.83) 0	0.9319	0.16 (−0.91, 1.23)	0.7786
Patients without heart failure (*N* = 153)	−0.58 (−0.87, −0.30)	<0.0001	−0.55 (−0.86, −0.24)	0.0006
LAVmax BSA				
Total patients (*N* = 170)	−0.02 (−0.31, 0.27)	0.8830	0.04 (−0.27, 0.35)	0.8200
Patients with heart failure (*N* = 17)	−0.68 (−1.87, 0.51)	0.2838	0.23 (−0.81, 1.27)	0.6738
Patients without heart failure (*N* = 153)	0.03 (−0.27, 0.32)	0.8607	0.05 (−0.27, 0.37)	0.7401
LAVmin BSA				
Total patients (*N* = 170)	0.14 (−0.10, 0.38)	0.2475	0.18 (−0.08, 0.44)	0.1837
Patients with heart failure (*N* = 17)	−0.35 (−1.38, 0.69)	0.5236	0.45 (−0.52, 1.42)	0.3953
Patients without heart failure (*N* = 153)	0.19 (−0.06, 0.43)	0.1346	0.19 (−0.08, 0.45)	0.1637

Model 1, adjusted for age, gender, ANP and BNP.

Model 2, adjusted for age, gender, smoking, alcohol consumption, eGFR, ANP, and BNP.

LVEF, left ventricular ejection fraction; LAV/BSA, left atrial volume/body surface area; eGFR, estimated glomerular filtration rate; ANP, atrial natriuretic peptide; BNP, brain natriuretic peptide.

### Subgroup analysis

We additionally performed subgroup analysis to calculate correlation coefficients for the association between BMI and LVEF, stratified by medical history, including hypertension and diabetes mellitus (as shown in Table 4). Similar statistical differences were observed in the patients with hypertension [correlation coefficient = −0.6; 95% CI: (−1.2, −0.1), *p* = 0.026] and those without hypertension [correlation coefficient = −0.7; 95% CI: (−1.2, −0.2), *p* = 0.009], after adjusting for age, sex, smoking, alcohol consumption, and eGFR. Interestingly, we also observed that higher BMI was negatively associated with lower LVEF in the AF patients without diabetes mellitus [correlation coefficient = −0.7; 95% CI: (−1.0, −0.3), *p* < 0.001] but not in those with diabetes mellitus [correlation coefficient = −1.3; 95% CI: (−3.8, 1.2), *p* = 0.353].

**Table 4:** The stratified analysis for association between BMI on admission and LVEF in AF patients.

Covariates	Model 1	
	Correlation coefficient (95% CI)	*p*-value
Hypertension		
Yes (*n* = 72)	−0.6 (−1.2, −0.1)	0.026
No (*n* = 98)	−0.7 (−1.2, −0.2)	0.009
Diabetes mellitus		
Yes (*n* = 13)	−1.3 (−3.8, 1.2)	0.353
No (*n* = 157)	−0.7 (−1.0, −0.3)	<0.001

Model 1, adjusted for age, gender, smoking, alcohol consumption and eGFR.

BMI, body mass index; AF, atrial fibrillation; LVEF, left ventricular ejection fraction; eGFR, estimated glomerular filtration rate.

## Discussion

Extensive literature has reported contradictory findings regarding the association between obesity and AF progression (5–7, 14–20). For example, a meta-analysis of 29 prospective studies showed that higher BMI or obesity was associated with an increased risk of AF (18). Similarly, a prospective cohort study from the United Kingdom reported that baseline BMI was related to a higher risk of developing AF (19). The recent Kailuan Study, which included a total of 44,135 participants from China, reported that elevated levels of BMI and waist circumference were associated with an increased risk of AF during a mean follow-up of 9.68 years (20). In contrast, some previous studies have suggested that overweight or obesity is associated with a significantly reduced risk of mortality and other adverse events (5–7). In our study, we observed that higher BMI was significantly associated with lower LVEF in the AF patients. However, there was no significant association between BMI and LAV in these patients.

Although the mechanisms underlying the “obesity paradox” in AF patients are not well understood, several possible explanations have been proposed, such as unknown confounding factors or a genuine biological phenomenon. First, age is an important factor influencing all-cause mortality and adverse events in AF patients (21). However, in most previous observational cohorts, AF patients with normal BMI tended to be older than those with higher BMI, and statistical adjustment cannot completely eliminate the confounding effect of age. Second, many existing studies have reported significant differences in AF management strategies, including rhythm control and anticoagulant use, among normal-weight, overweight, and obese patients (22, 23). This highlights the complexity of managing AF, which involves many clinical and pathological factors. Third, obesity might be related to differences in nutritional status (24), and the “obesity paradox” has not been consistently observed for adverse clinical outcomes, including heart failure and stroke, among AF patients (22, 23, 25). For instance, a previous study including a total of 2,592 patients with non-valvular AF from 35 centers in Turkey divided participants into two groups: 761 patients who died and 1831 patients who survived (25). The study concluded that malnutrition, assessed using three scoring systems, was an independent predictor of all-cause mortality in these AF patients (25). Finally, some scholars have argued that this apparent paradox might be the result of selection bias or other paradoxes (26, 27). Interestingly, our results showed that the independent relationship between BMI and LVEF was not observed in patients with heart failure. Several factors may explain this phenomenon. On one hand, heart failure itself is a potential confounding factor that can lead to insignificant results, producing completely opposite trends between patients with and without heart failure. On the other hand, the small number of patients with heart failure in our study limits the statistical power, making it difficult to draw definitive conclusions. Therefore, further evidence from larger, multicenter studies is needed to clarify this association.

Our study has several strengths. First, we found that higher BMI was related to reduced LVEF in the AF patients. However, there was no significant association between BMI and LAV/BAS in these patients, suggesting that our results might have important implications for public health policies regarding AF prognosis, indicating that obesity may be related to deteriorating cardiac systolic function in AF patients. Second, BMI was calculated from directly measured weight and height rather than self-reported data. Third, this is the first study to investigate the association between BMI and LVEF in AF patients using subgroup analysis. We also acknowledge several limitations in our study. First, a causal relationship between BMI and LVEF in the AF patients could not be established because of the inherent nature of the cross-sectional analysis. Second, different types or severities of AF have different prognoses. However, we were unable to confirm AF type in this study due to its retrospective design. In addition, the LVEF of all included patients was more than 35%, and individuals with LVEF below 35% were not analyzed because of the retrospective analysis. Third, as this was a single-center study, the generalizability of our findings is limited. Fourth, the results would be more reliable if additional parameters, such as waist-to-height and waist-to-hip ratios, had been included. Fifth, measured BMI value in our study was obtained from only one time, thus underestimated variability for BMI may be existed. Sixth, the small sample size for certain subgroups (e.g., heart failure patients) and insufficient confounding factors were conducted, which needed multi-center and prospective evidence to validate.

## Conclusion

In brief, our study demonstrated that higher BMI was strongly associated with lower LVEF but not with LAV/BSA in the obese patients with AF. These findings may provide new insights into the association between obesity and cardiac function in individuals with AF. Further evidence from larger, multicenter studies is needed to confirm our results.

## Data Availability

The raw data supporting the conclusions of this article will be made available by the authors, without undue reservation.

## Footnotes

1
https://datadryad.org/stash/dataset/doi:10.5061/dryad.869b2n0
